# Supplementary material for: Vaccine-Induced T-Cell and Antibody Responses at 12 Months after Full Vaccination Differ with Respect to Omicron Recognition
Source: Vaccines (Basel). 2022 Sep 19;10(9):1563. doi: 10.3390/vaccines10091563 (PMC9500953; doi:10.3390/vaccines10091563)
Supplement: Supplementary file 1 [file vaccines-10-01563-s001.zip › vaccines-1898885-supplementary.pdf]

vaccines

MDPI

Supplementary materials

Vaccine-induced T- and B-cell responses differ with respect to Omicron recognition

Franz Mai [1], Johann Volzke [1], Emil C. Reisinger [2] and Brigitte Müller-Hilke [1,*]

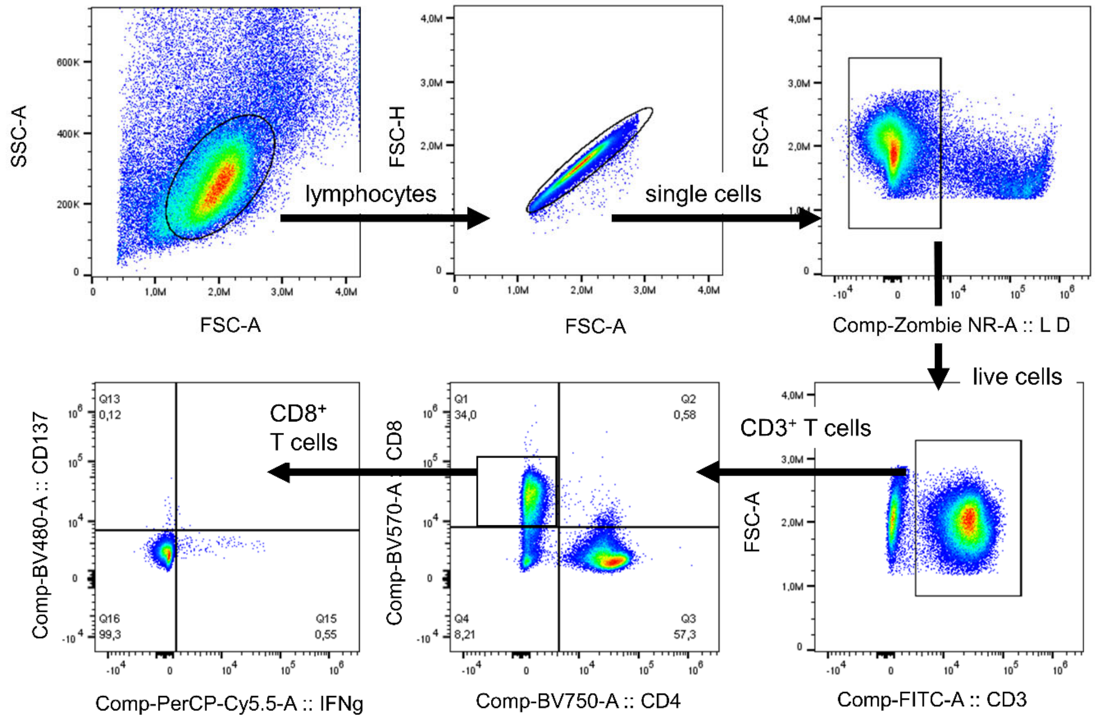

Figure S1. Gating scheme for analyzing T cell responses following BNT162b2 re-stimulation. Arrows indicate hierarchical gating steps.

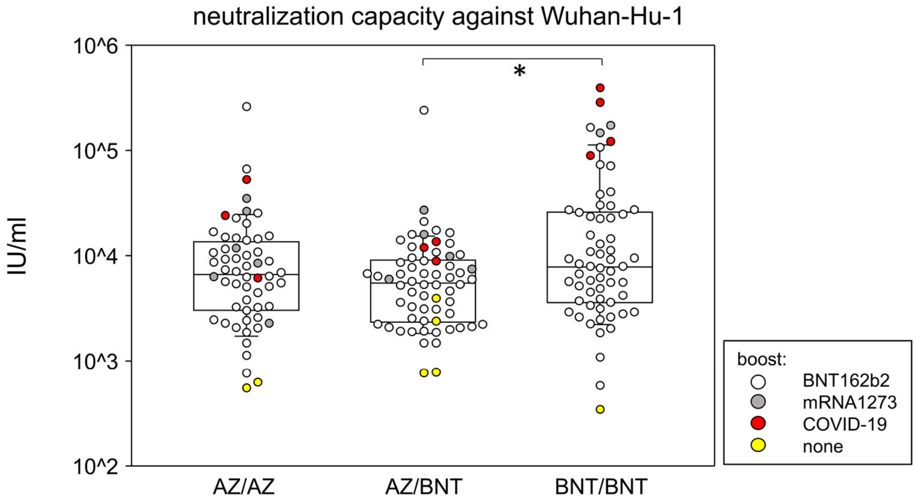

Figure S2. Primary / secondary immunization with BNT162b2 and hybrid immunity led to maximum neutralization capacities against the Wuhan-Hu-1 spike protein. Dot plots and corresponding box plots show neutralization capacities against the Wu-Hu-1 spike protein presented in IU/ml. AZ/AZ: homologous primary/secondary immunization with AZD1222, AZ/BNT: heterologous primary/secondary immunization with AZD1222 and BNT162b2, BNT/BNT: homologous primary/secondary immunization with BNT162b2. Color codes indicate boost variants. Kruskal-Wallis test followed by Dunn´s multiple comparison test resulted in a p value of 0.0180, asterisk indicates statistically significant differences.

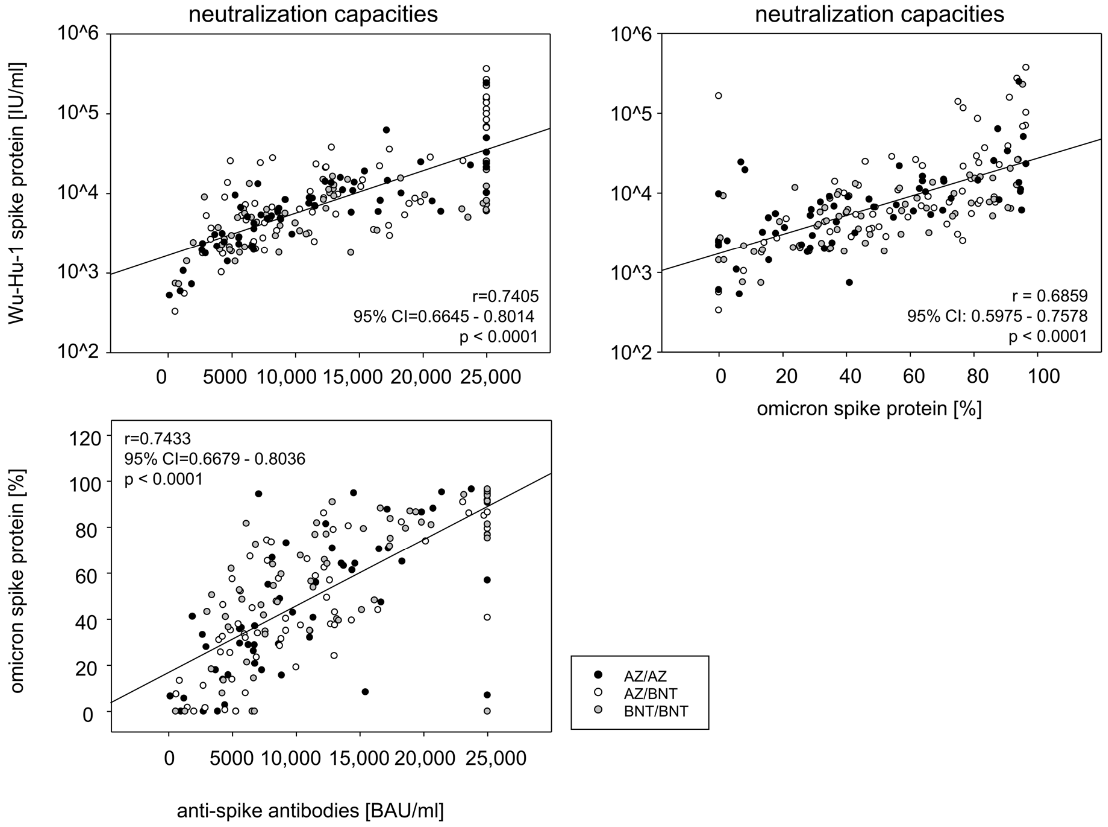

Figure S3. Correlation between antibody concentration and neutralization capacities. Dot blots show the correlation between anti-spike antibody concentrations (BAU/ml) and their neutralization capacities against the wild type (Wu-Hu-1) spike protein (upper left panel) and its Omicron variant (lower left panel) and the correlation between neutralization capacities against Wu-Hu-1 and Omicron (upper right panel). Correlation coefficients (r), 95% confidence intervals (CI) and p values result from Spearman rank correlation analyses. The color scheme indicates vaccination regimen.

Table S1. T cell response to *in vitro* re-stimulation with BNT162b2

			**CD4$^+$ T helper cells (n/10^6 live PBMC)**						**CD8$^+$ cytotoxic T cells (n/10^6 live PBMC)**					
**Probe**	**BAU/ml**	**IU/ml**	**CD137$^+$**	**IL-2$^+$**	**IFNγ^+**	**TNFα^+**	**IL-4$^+$**	**IL-10$^+$**	**CD137$^+$**	**IL-2$^+$**	**IFNγ^+**	**TNFα^+**	**IL-4$^+$**	**IL-10$^+$**
A03	1870	734	381	488	548	71	1202	381	488	1381	2060	381	464	405
A07	6729	2002	557	1437	356	223	323	189	490	646	1158	290	345	234
A09	6649	2196	325	560	347	168	336	403	358	493	437	157	90	604
A17	12344	14458	249	554	158	170	238	34	238	724	1063	769	215	68
A20	13719	11362	603	1346	81	46	313	858	464	870	522	429	452	487
A21	6766	3653	266	578	393	243	705	312	497	1086	566	439	243	578
A23	16664	8326	848	218	344	103	115	401	527	241	860	252	149	447
A24	13547	16199	247	82	141	141	634	258	646	164	693	423	493	399
A25	11101	9069	343	1803	245	98	417	601	527	1337	1288	343	196	552
A45	2672	1982	591	230	591	115	230	1673	1214	2789	1034	574	164	771
AB05	12916	16762	635	1019	192	372	240	456	971	468	1391	360	432	492
AB06	13033	9529	718	581	251	171	274	388	980	570	718	137	114	376
AB14	4860	1989	341	1139	223	352	317	1843	282	1033	505	481	223	1949
AB16	24974	6379	868	1505	339	54	285	95	596	664	2006	529	230	366
AB18	12704	10382	733	577	666	78	533	766	555	355	1199	477	56	522
AB20	10350	3449	498	815	1222	106	709	785	1494	1283	5387	2807	2007	1343

AB21	6891	2037	382	111	731	64	366	334	906	700	1097	572	207	811
AB36_COV	6390	13550	525	110	171	147	342	489	794	440	1417	171	195	525
AB50	10863	3141	694	0	256	97	475	450	1351	402	1764	110	986	377
AB51	8060	5093	474	71	391	296	273	427	569	498	948	569	225	427
AB53	13020	13070	289	185	369	173	600	808	635	162	658	173	150	381
AB54_COV	2873	9171	257	315	432	128	350	595	665	257	1109	432	607	502
B24	6668	2378	725	456	362	188	268	443	416	523	456	174	456	376
B25	17397	3020	423	833	579	85	97	785	483	857	1050	664	97	736
B35_C0V	25000	117376	457	169	265	72	361	445	313	337	1420	301	241	481
B41	7579	1954	164	197	181	82	756	986	2021	592	2432	312	181	1068
B44	25000	103092	776	418	1239	90	448	2538	941	418	836	299	254	747
B47	6750	2515	589	360	589	147	671	1260	1391	376	1521	703	147	753
B54	8227	28754	265	1236	177	99	254	177	530	839	1435	143	320	342
B55	6562	2161	458	153	305	127	662	598	1387	1450	2392	305	1120	382
B56	11493	2506	139	506	367	127	203	342	1140	810	1405	329	696	532
B57	3353	2693	1117	444	279	114	355	673	178	685	584	114	216	203
B58	4194	1042	757	244	98	12	671	183	1745	793	1074	73	195	598
B62_COV	25000	378653	859	163	218	239	370	196	2164	729	631	381	141	620

A : homologous primary / secondary immunization with AZD1222; AB: heterologous primary / secondary immunization with AZD1222/BNT162b2; B: homologous primary / secondary immunization with BNT162b2. _COV: infection withSARS-CoV-2 instead of mRNA boost.

Table S2. Restimulation with Wu-Hu-1 and Omicron peptide pools did not show significantly increased expression of the following markers

	without stimulation positive cells /10^6 live PBMC [median]	Wu-Hu-1 stimulated positive cells /10^6 live PBMC [median]	Omicron stimulated positive cells /10^6 live PBMC [median]	Comparison w/o stimulation vs. Wu-Hu-1 [P]*	Comparison w/o stimulation vs. Omicron [P]*
CD4+ T-Cells expressing					
CD25	18,774	22,287	21,386	0.327	0.779
CD137	462	363	415	0.889	0.889
Fas-L	2,381	2,691	2,601	0.889	0.263
TNFα	109	139	95	0.263	0.575
IL-4	188	291	262	0.674	0.779
IL-10	518	1,146	1,212	0.161	0.327
GrzB	27,399	25,341	24,223	0.779	0.327
CD8+ T-Cells expressing					
CD25	92,122	107,849	96,503	0.208	0.401
CD137	949	920	970	0.779	0.889
Fas-L	1,175	950	842	0.575	0.779
IL-2	624	681	646	0.263	0.263
IFNγ	1,198	950	785	0.779	0.575
TNFα	499	598	320	0.779	0.208
IL-4	197	129	128	0.327	0.128
IL-10	604	573	597	0.208	0.327
GrzB	110,578	113,903	110,247	1	0.484

* Wilcoxon matched pairs signed rank test
